# An HLA Association With COVID-19 Vaccine Reactogenicity Correlates With Fewer SARS-CoV-2 Infections and Monocyte Activation

**DOI:** 10.21203/rs.3.rs-8282930/v1

**Published:** 2025-12-17

**Authors:** Jill Hollenbach, Anshika Srivastava, Demetra Chatzileontiadou, Anurag Adhikari, Rayo Suseno, Sean Lin, Juliano Boquett, Jamie Tuibeo, Tasneem Yusufali, Noah Peyser, Ticiana Farias, Katherine Kichula, Andrea Nguyen, Irvin Jose, Dhilshan Jayasinghe, Katerina Tarassi, Elissavet Kontou, Janesha Maddumage, Kleio Ampelakiotou, Alexandra Tsirogianni, Michael Dewar-Oldis, Peter Barnard, Joe Sabatino, Dimitrios Zoulas, Emily Ariens, Timothy Mercer, Emma Grant, Lloyd D’Orsogna, Corey Smith, Paul Norman, Gregory Marcus, Jeffrey Olgin, Mark J. Pletcher, Martin Maiers, Stephanie Gras

**Affiliations:** University of California San Francisco; University of California San Francisco; Monash University; Immunity and Infection Program, La Trobe Institute for Molecular Science (LIMS), La Trobe University; University of California San Francisco; University of California San Francisco; University of California San Francisco; Immunity and Infection Program, La Trobe Institute for Molecular Science (LIMS), La Trobe University,; University of California San Francisco; University of California San Francisco; University of Colorado School of Medicine,; University of North Carolina at Charlotte; 2. Immunity and Infection Program, La Trobe Institute for Molecular Science (LIMS), La Trobe University,; Immunity and Infection Program, La Trobe Institute for Molecular Science (LIMS), La Trobe University,; Immunity and Infection Program, La Trobe Institute for Molecular Science (LIMS), La Trobe University,; Blood Bank Department, “Evangelismos” General Hospital,; Immunology-Histocompatibility Department, “Evangelismos” General Hospital; Immunity and Infection Program, La Trobe Institute for Molecular Science (LIMS), La Trobe University,; Blood Bank Department, “Evangelismos” General Hospital,; Blood Bank Department, “Evangelismos” General Hospital,; Immunity and Infection Program, La Trobe Institute for Molecular Science (LIMS), La Trobe University,; La Trobe university; Department of Neurology, University of California,; Immunity and Infection Program, La Trobe Institute for Molecular Science (LIMS), La Trobe University,; BASE mRNA Facility, Australian Institute for Bioengineering and Nanotechnology (AIBN), University of Queensland,; University of Queensland; Immunity and Infection Program, La Trobe Institute for Molecular Science (LIMS), La Trobe University,; University of Western Australia; QIMR Berghofer Medical Research Institute; University of Colorado, Denver; Division of Cardiology, University of California, San Francisco; UCSF; UCSF; National Marrow Donor Program; La Trobe University

## Abstract

Vaccination against SARS-CoV-2 has been the most effective tool in mitigating the COVID-19 pandemic. However, some individuals experience side effects that cause distress and interfere with daily activities, which can limit vaccine uptake, with important public health implications. Here, we considered the impact of HLA variation on the propensity for mild side effects with COVID-19 vaccination. We examined variation in HLA-A, -B, -C, -DRB1, and -DQB1 for association with self-reported side effects in a large cohort of U.S. Euro-ancestry vaccinated individuals (N = 50,535) and confirmed results in an independent replication cohort (N = 4,575). We found that HLA-A*03:01 was significantly associated with systemic side effects (OR = 1.36, CI = 1.31–1.41, p = 6.79×10–57) and fewer breakthrough infections, and that this phenomenon is specific to the COVID-19 vaccine. Surprisingly, we observed limited activation of CD8+ T cells in HLA-A*03:01+ samples to the Spike-derived peptides, excluding them as a likely source of the reported vaccine side effects. Rather, examination of immune cell subsets, prior and after vaccination, points to a central role for monocytes in the production of IL-6 and IL-8, which significantly correlates with the reported severity of side effects in HLA-A*03:01+ donors. Meanwhile, the large, mostly naïve, and low-affinity population of Spike-specific CD8+ T cells likely contribute to an inflammatory milieu in HLA-A*03:01 carriers through weak binding to antigen presenting cells. This work sheds light on the mechanisms underlying HLA-mediated COVID-19 vaccine reactogenicity and associated reduction in infections, providing important new insights that may support efforts to optimize vaccine efficacy and promote broader public involvement in vaccination programs.

## Introduction

COVID-19 vaccines are an important public health tool in preventing severe illness, hospitalization, and mortality due to infection with the virus^1–4^. In early studies examining the effectiveness of these vaccines, mRNA vaccines BNT162b2 (referred to hereafter by the brand name “Pfizer”) and mRNA-1273 (referred to hereafter by the brand name “Moderna”) were shown to be 95% and 94.1% effective against symptomatic infection, respectively^5^. Adenovirus-based vaccines, such as ChAdOx1-S (brand name “Johnson & Johnson”, hereafter “J&J) and Ad26.COV2.S (brand name “AstraZeneca”), in contrast, showed 70% and 66% efficacy, respectively^6,7^. Thus, although mostly efficacious in preventing serious illness and hospitalization, ‘breakthrough’ infections (BTI) occur with all current COVID-19 vaccines. These infections may be attributed to primary vaccine failure, secondary vaccine failure, individuals’ age, immune evasion by novel viral variant, or waning vaccine efficacy over time^8–15^.

Prior work has indicated that the immunogenicity of the COVID-19 vaccines can vary by individual and is correlated with their efficacy. For example, studies have reported different immunological responses to vaccines linked to age, sex, body mass index, nutritional status, and the composition of the gut microbiome^16,17^. Suggesting an immunogenetic feature of vaccine response, several studies have linked variation in the human leukocyte antigen (*HLA*) region with antibody levels and T-cell response after vaccination^18–21^. *HLA* is the most polymorphic region (6p21) of the human genome with thousands of known alleles. Variation in *HLA* is long recognized to play a role in viral illness^22^. Demonstrating the importance of HLA antigen presentation in the immune response to SARS-CoV-2, we have previously shown that *HLA* variation is associated with an asymptomatic disease course^23^ and provided a functional and structural basis to explain the association. Likewise, numerous other *HLA* associations with the COVID-19 disease course have been identified^24^.

While overwhelmingly safe, vaccination-induced immune activation can lead to side effects. Most adverse reactions are mild, including fever, muscle aches, headaches, and fatigue, as well as local reactions such as pain, redness, and swelling at the injection site^25^. These reactions typically appear within a few hours after vaccination and are short-lived, usually resolving within one to two days^26^. While bothersome, there is some evidence that side effects may be associated with improved vaccine efficacy. A study involving participants receiving the human papillomavirus vaccine, for example, found that the occurrence of inflammation-related adverse reactions is associated with concentrations of antibodies, suggesting that individuals who have vaccine-induced side effects have a more robust immune response^27,28^. Importantly, systemic side effects from COVID-19 vaccination, such as fever and fatigue, have also been associated with enhanced humoral and cellular immune responses^29^. Intriguingly, previous work has shown a specific *HLA* class I allele, HLA-A*03:01 to be associated with increased side effects after COVID-19 vaccination as well as increased antibody response^19,30,31^. Additionally, enhancement of T cell memory from COVID-19 mRNA booster doses was shown to be particularly pronounced in HLA-A*03:01^+^ COVID-19 recovered patients^20^.

Nevertheless, despite strong evidence for the role of HLA in response to COVID-19 vaccination, there remains substantial knowledge gaps regarding the complex interplay between *HLA* genetic variation, HLA structural variation and antigen presentation, vaccine side effects, cellular and humoral immunity, and vaccine effectiveness. In the present study, we close these gaps by considering *HLA* variation and vaccine reactogenicity in a large cohort of over 50,000 vaccinated subjects and provide a global framework for our findings by examining differential gene expression, antibody response, T cell reactivity, and innate immunity. We demonstrate that HLA-A*03:01-associated reactogenicity is associated with fewer total infections and is not driven by T cell activation, despite a large pool of mostly naïve Spike-specific T cells, but rather by monocyte-derived cytokine production, revealing an innate immune mechanism underlying HLA-linked COVID-19 vaccine side effects.

## Results

### Participant recruitment and baseline demographics

Between January 2023 and January 2024, we collected responses to survey questions regarding respondents’ general health history, including experiences with COVID-19 and associated vaccinations, from potential bone marrow donors registered with the NMDP (formerly National Marrow Donor Program/Be The Match) and for whom high-resolution *HLA* genotyping data were available in the NMDP database. The specific survey questions related to COVID-19 and vaccinations, including side effects queried, are given in **Supplementary Table 1**. Because this was a U.S.-based cohort, respondents received only vaccines approved for use in the country (Pfizer, Moderna, J&J). Among the 80,007 total respondents, 50,535 self-identified as White/European ancestry (**Supplementary Table 2a**) and reported having completed at a minimum the initial series of vaccination for SARS-CoV-2 (one dose for J&J, two for Pfizer/Moderna mRNA); these subjects constituted our discovery cohort. An additional 7403 respondents reported completion of the initial series self-identified with other ancestries (**Supplementary Table 2b**).

For our replication cohort, we collected responsesbetween July 2020 and April 2022 via a mobile phone app with follow-up daily questions specific to vaccine side effects, as described in Augusto et al., 2023^23^. Among 10,595 respondents who reported completion of the initial vaccination series, 4,575 self-identified as White/European ancestry (**Supplementary Table 3**). While these respondents are also NMDP donors with available high resolution *HLA* data, there was no overlap with individuals in the discovery cohort.

### Systemic side effects to COVID-19 vaccines co-occur and are associated with HLA-A*03:01

To understand the distribution of side effects to COVID-19 vaccines in our discovery cohort, we first calculated the covariation matrix for all reported side effects (**Supplementary Figure 1**). Here, we only considered side effects reported after completion of the initial vaccination series. We found that systemic side effects (SSE) like fever or chills, muscle or body fatigue, or headaches, rather than localized side effects like runny nose (**Supplementary Table 4**), showed substantial co-occurrence, with a median number of two SSE reported per individual.

**Figure 1:**
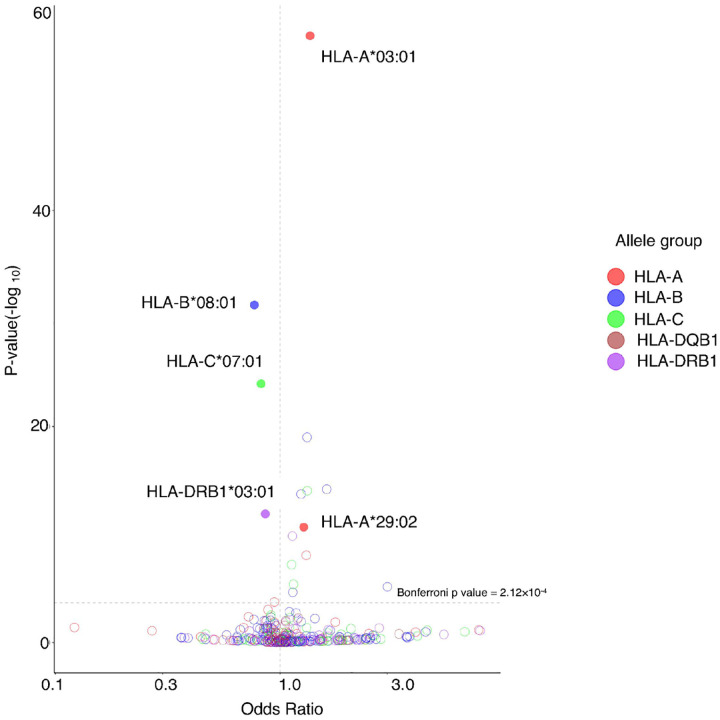
Association of *HLA* alleles with three or more systemic side effects in the discovery cohort (Europeanancestry, N = 50,535). Odds ratio is shown on the x-axis and p-value is shown on the y-axis. Alleles that are significant in both discovery and replication cohort are shown as solid-colored circles.

To evaluate whether HLA variation plays a role in increasing side effects in vaccination for SARS-CoV-2 and to capture cases with the greatest burden of symptoms, we first considered individuals who reported greater than the median number of SSE. Using a dominant model, we used multivariate logistic regression to test for association with the occurrence of three or four reported SSE for each *HLA* allele at each classical class I (*HLA-A, -B, -C*) and two *HLA* class II (*HLA-DRB1* and -*DQB1*) loci observed at a frequency >3% in our cohort, adjusting for sex and age (Figure 1, **Supplementary Table 5**). This revealed as the top candidate a strong and significant association of HLA-A*03:01 with SSE (OR = 1.36, CI= 1.31 – 1.41, p = 6.79×10^−57^). We did not observe any substantial dose effect for HLA-A*03:01 (homozygous, OR = 1.47, CI = 1.29 – 1.68, p = 1.65×10^−8^; heterozygous, OR = 1.40, CI = 1.34 – 1.46, p = 3.15 ×10^−53^), confirming the dominant model. Because HLA-A*03:01 belongs to the HLA-A3 supertype group^32^ that also includes HLA-A*11:01, -A*31:01, -A*33:01, and -A*68:01, and is characterized by shared peptide binding^33^, we hypothesized that this supertype might show shared associations with SSE across alleles. However, we found that among the HLA-A3 supertype alleles, only HLA-A*03:01 was significant for the association with increased SSE (**Supplementary Table 5**). This association of increased SSE with HLA-A*03:01 clearly replicated in our additional cohort of 4,575 vaccinees with European ancestry, with a remarkably consistent effect size (OR = 1.46, CI = 1.21 – 1.77, p = 7.77 × 10^−5^) relative to that in our discovery cohort (**Supplementary Table 6)**. In addition, we found that HLA-A*03:01 was significantly associated with increased SSE reports in our self-identified Hispanic cohort (N = 4,287), again with extremely consistent effect size (OR = 1.45, CI = 1.25 – 1.69, p = 7.31× 10^−7^, **Supplementary Table 7**). While a similar trend was clearly observed, this association did not reach statistical significance in cohorts of other ancestries, where our sample sizes were much smaller (**Supplementary Tables 8** and **9**).

To better understand whether any specific reported side effect was driving this HLA association, we considered the association with HLA-A*03:01 for each side effect separately (**Supplementary Table 10**). As expected, we observed a strong and highly significant negative association of HLA-A*03:01 with reporting “no vaccine side effects” (OR = 0.73, CI = 0.70 – 0.77, p = 5.82×10^−43^), demonstrating that individuals with this allele are less likely to report having experienced no side effects after vaccination. Analysis of reports of specific side effects revealed the strongest association of HLA-A*03:01 with “fever or chills” (OR = 1.43, CI = 1.38 – 1.49, p = 1.37×10^−81^), followed by “muscle or body aches” (OR = 1.305, CI = 1.25 – 1.35, p = 2.16 ×10^−45^), “fatigue” (OR = 1.25, CI = 1.20 – 1.30, p = 5.57×10^−34^), and “headaches” (OR = 1.23, CI = 1.19 – 1.29, p = 1.00×10^−24^). We also found remarkably consistent results for our cohort of individuals who self-identify as Hispanic (**Supplementary Table 10**, **Supplementary Figure 2**). Only “fatigue” was found to have a significant association with HLA-A*03:01 in our African American cohort (OR = 1.49, CI = 1.07 – 2.09, p = 0.018); however, as noted previously, smaller sample sizes limited our power to detect associations in some cohorts (**Supplementary Table 8** and **9**). Finally, we did not find a substantive difference in effect size when considering individuals who had reported infection prior to vaccination or breakthrough infection (BTI)(**Supplementary Table 11**).

In summary, we find a highly significant, consistent association of HLA-A*03:01 with reported side effects post-COVID-19 vaccination. This association replicated across multiple independent cohorts and ancestries, supporting a role for *HLA* variation in driving vaccination side effect.

### Effect size of HLA-A*03:01 varies with vaccine brand

While the median number of SSE reported across the entire discovery cohort was two, the proportion of individuals who experienced three or four SSE was higher in Moderna than in Pfizer or Johnson & Johnson vaccinated individuals (**Supplementary Figure 3**). Because the frequency of SSE varied by vaccine manufacturer, we stratified our cohorts according to whether they received the Pfizer, Moderna, or J&J vaccine. To reduce confounding, we only considered individuals who received both doses of the initial series (in the case of mRNA vaccines) from the same manufacturer.

We found that Pfizer-vaccinated individuals showed the largest effect size for all associations of HLA-A*03:01 with side effects relative to other vaccine brands, particularly fever or chills (OR = 1.71, CI = 1.61 – 1.79, p = 4.51×10^−88^). Likewise, the chance of not experiencing any side effects is significantly lower in Pfizer (OR = 0.676, CI = 0.64 – 0.71, p = 2.83×10^−40^) and Moderna recipients (OR = 0.782, CI = 0.72 – 0.85, p =1.59×10^−9^) (**Supplementary Table 12**). We did not observe significant associations of HLA-A*03:01 with side effects among J&J vaccine recipients except for fatigue (OR = 1.259, CI = 1.09 – 1.45, p = 1.40×10^−4^), possibly owing to the small sample size for this brand (N = 3,312). We observed similar results in our replication cohort, where the association of HLA-A*03:01 showed similar patterns of effect size between brands, although many individual side effects did not reach statistical significance among Moderna recipients, likely due to more limited power in this cohort (**Supplementary Table 13**).

Thus, while we find that HLA-A*03:01 is associated with side effects across COVID-19 vaccine brands, this effect is much more pronounced and consistent among Pfizer recipients.

### Additional HLA class I alleles are associated with systemic side effects in COVID-19 vaccination

In addition to HLA-A*03:01, we observed numerous other *HLA* alleles that were significantly associated with reports of systemic side effects in our discovery cohort (Figure 1, **Supplementary Table 5**). Of these, HLA-A*29:02 (OR = 1.28, CI = 1.19 – 1.36, p = 2.10×10^−11^), HLA-B*08:01 (OR = 0.76, CI = 0.73 – 0.80, p = 5.65×10^−32^), HLA-C*07:01 (OR = 0.82, CI = 0.79 – 0.85, p = 1.10×10^−24^), and *HLA*-DRB1*03:01 (OR = 0.85, CI = 0.82–0.90, p = 1.23×10^−12^) replicated (**Supplementary Table 6**). HLA-B*08:01, HLA-C*07:01, and HLA-DRB1*03:01 are components of the well-documented “ancestral 8.1 haplotype” (AH8.1)^34^, which is also evident from the high linkage disequilibrium (LD) values between these alleles (**Supplementary Table 14**). Owing to the likelihood that these three alleles reflected a single primary association through LD, we performed conditional analyses to identify the primary associated allele in this haplotype. We found that HLA-B*08:01 had the strongest effect size (OR = 0.77, CI = 0.71 – 0.84, p = 2.71×10^−9^) after controlling for HLA-C*07:01, and HLA-DRB1*03:01, suggesting that this allele is responsible for the protective effect against SSE. Likely owing to the small sample size, we did not detect these associations in our cohorts with other ancestries (**Supplementary Tables 7 – 9**).

In summary, while HLA-A*03:01 demonstrated the strongest and most significant effect with respect to vaccine SSE, we find evidence for additional HLA class I involvement for both risk (HLA-A*29:02) and protection (HLA-B*08:01).

### HLA-A*03:01 carriage is associated with higher antibody levels after COVID-19 vaccination

We next sought to confirm prior reports of association of HLA-A*03:01 with higher levels of antibody upon vaccination for SARS-CoV-2^19,35,36^ in an independent cohort of 156 healthcare workers receiving two doses of the Pfizer vaccine. The cohort was stratified in three groups according to the levels of anti-Spike IgG antibodies after the second dose of the vaccine as follows, Group I: 1,000 – 4,000 AU/mL (N = 50), Group II: 4,001 – 20,000 AU/mL (N = 53), Group III: ≥ 20,000 AU/mL (N = 53). To investigate the association between HLA-A*03:01 and anti-Spike antibody levels, we performed logistic regression comparing Group III against Groups I and II. Our results confirmed that HLA-A*03:01 is associated with higher antibody levels post vaccination (OR = 3.25, CI = 1.31 – 8.33, p = 0.0117). We also observed that antibody positivity increased with HLA-A*03:01 allele dosage (0, 1, or 2 copies; **Supplementary Figure 4**). Thus, we confirm that in addition to its role in mediating vaccine SSE, individuals positive for HLA-A*03:01 display a higher anti-Spike antibody response post-vaccine, providing further evidence that vaccine side effects are reflective of a more robust immune response to vaccination and that this has an immunogenetic underpinning.

### HLA-A*03:01 carriers report fewer breakthrough infections, fewer total infections, and milder disease course

Owing to the highly significant association of HLA-A*03:01 with vaccine side effects and increased antibody levels after vaccination, we next asked whether this allele might be associated with reduced breakthrough infection (BTI). Of the total vaccinated 57,938 individuals across ancestries, we excluded 10,227 individuals who did not have information for their 2^nd^ dose timing. Among the remaining participants who reported specific dates for both vaccination and infection, we found HLA-A*03:01 to be associated with a decreased risk of BTI, albeit with modest effect size (OR = 0.916, CI = 0.88 – 0.95, p = 1.61×10^−5,^); this association was also significant with stronger effect size in our cohort with Hispanic ancestry (OR = 0.776, CI = 0.66 – 0.91, p = 1.44×10^−4^), and while not reaching statistical significance, we observed similar effect sizes in other ancestries(**Supplementary Table 15**). We did not observe any other significant HLA associations with BTI in our cohort, including previously reported associations of HLA-DQB1*06^18,37^.

The observed association of HLA-A*03:01 with reduction in BTI is also reflected in an overall decrease in total infections reported. We dichotomized participants according to those reporting having never been infected or having only a single infection, (about 86% of our discovery cohort, N= 43,527), and those reporting repeated reinfections (i.e. two or more infections, about 14% of our discovery cohort, N = 7008) between 2019 and 2023. We found that vaccinated individuals positive for HLA-A*03:01 were less likely to have experienced repeated SARS-CoV-2 infections (OR = 0.92, CI = 0.87 – 0.96, p = 0.00177). Finally, we asked whether among those who did report infection, HLA-A*03:01 is associated with milder disease course. We found that individuals positive for HLA-A*03:01 were less likely to report higher than the median number (three) of symptoms (considering all reported symptoms; OR = 0.90, CI = 0.88 – 0.94, p = 2.78 × 10^−7^) suggesting a milder COVID-19 disease course.

To understand whether the HLA-A*03:01 association with reduced BTI was secondary to its association with side effects, we considered the occurrence of vaccination side effects and the likelihood of BTI irrespective of *HLA* genotype. We found that each systemic side effect **was** independently inversely associated with the likelihood of a BTI (**Supplementary Table 16**). Considering the combined measure of three or four SSE and BTI (and adjusting for sex, age, and vaccine brand), we found a highly significant protective effect with an effect size greater than that observed for HLA-A*03:01 (OR = 0.83, CI = 0.79 – 0.87, p = 6.03×10^−14^). When we include HLA-A*03:01 as a covariate in our model, we observed a nearly identical association of SSE with BTI (OR = 0.82, CI = 0.79 – 0.87, p = 9.40×10^−14^), while the HLA-A*03:01 (OR = 0.93, CI = 0.89 – 0.98, p = 5.81×10^−3^) association was only borderline significant, suggesting an association independent from *HLA*. Finally, we stratified our cohort according to the carriage of HLA-A*03:01 and further observed a highly significant and consistent inverse association between the combined measure for three or four SSE and the risk of BTI in individuals without the HLA-A*03:01 allele (OR = 0.81 CI = 0.78 – 0.85, p = 4.32×10^−19^). Thus, we conclude that the negative association of HLA-A*03:01 with BTI stems from its role in increasing vaccine reactogenicity, along with a global negative association of vaccine side effects with BTI.

### HLA-A*03:01 associated vaccine reactogenicity is specific to COVID-19 vaccines

To fully contextualize our findings, we sought to compare responses to COVID-19 vaccines with those of other widely administered vaccines, such as the currently approved influenza vaccine. We administered a follow-up questionnaire to the same individuals included in our discovery cohort (**Supplementary Table 17**). This survey specifically assessed the occurrence of influenza vaccine-associated side effects and the frequency with which participants received influenza vaccinations. As of April 2025, we received responses from a total of 13,499 participants from our discovery cohort, among whom 11,916 individuals reported having received the influenza vaccine at least once (**Supplementary Table 18**). Of those receiving the vaccine, 3,417 individuals reported experiencing side effects. As for the COVID-19 vaccine, we performed multivariate logistic regression to assess associations between each *HLA* allele and the presence of at least one side effect (**Supplementary Table 19**). None of the tested *HLA* alleles reached the Bonferroni-corrected threshold for statistical significance (p = 7.14 ×10^−4^), including HLA-A*03:01. Thus, the *HLA* association with vaccine reactogenicity appears to be specific to the COVID-19 vaccines.

Despite the lack of *HLA*-specific associations, we observed an epidemiologically meaningful correlation: individuals experiencing side effects following influenza vaccination were more than three times (OR = 3.094, CI = 2.73 – 3.50, p = 2 ×10^−16^) as likely to report side effects from the COVID-19 vaccine, after controlling with HLA-A*03:01. Thus, it appears that there are factors beyond *HLA* predisposing some individuals to experiencing vaccine reactogenicity.

### High frequency of low avidity Spike-specific T cell population is observed ex vivo

The strong association between HLA-A*03:01 carriage and the presence of side effects after COVID-19 vaccination, coupled with more robust immunity to the virus (higher Spike-specific antibody and less breakthrough infection occurrence), led us to hypothesize that HLA-A*03:01^+^ individuals could mount a strong and perhaps disproportionate CD8^+^ T cell response, as previously suggested by others^30^. To assess this hypothesis, blood from HLA-A*03:01^+^ and HLA-A*03:01^−^ individuals was collected, most without SARS-CoV-2 infection, prior (V0) and post vaccination with either 1 dose (V1) or 2 doses (V2) of the Pfizer or AstraZeneca vaccine (**Supplementary Table 20**). The peripheral blood mononuclear cells (PBMCs) and plasma were isolated and vaccination side-effects self-reported (converted to a side-effect severity score) (**Supplementary Table 21**).

**Figure 2:**
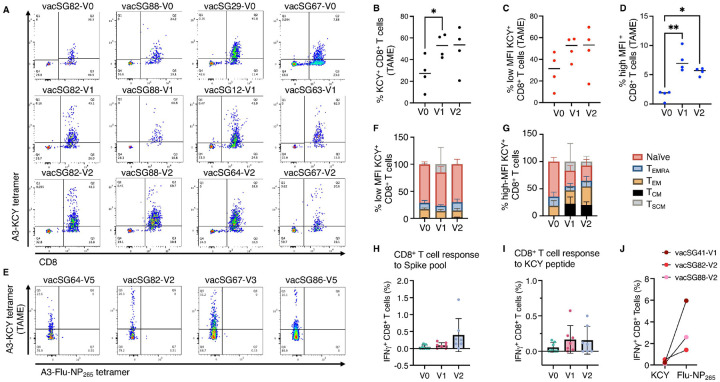
Spike-specific CD8^+^ T cells are present before vaccination and expand into a high-MFI effector population after vaccination. (**A**) Tetramer-associated magnetic enrichment (TAME) plots of HLA-A*03:01-KCY-specific CD8^+^ T cells across before (V0) and after vaccination (V1, V2). (**B**) Quantification of KCY^+^ CD8^+^ T cell frequencies by TAME. (**C-D**) Mean fluorescence intensity (MFI) analysis of HLA-A*03:01-KCY-specific CD8^+^ T cells with (**C**) low- and (**D**) high-MFI at the three time point V0, V1 and V2. (**E**) Double-tetramer staining of HLA-A*03:01-KCY-specific CD8^+^ T cells with HLA-A*03:01-Flu-NP_265_. (**F-G**) Phenotypic composition of (**F**) low-and (**G**) high-MFI HLA-A*03:01-KCY-specific CD8^+^ T cells showing the proportion of naïve (red), effector memory (TEM, orange), central memory (TCM, black), stem like memory cells (TSCM, grey), and terminally exhausted memory re-expressing RA cells (T_EMRA_, blue) phenotype. (**H-I**) IFNγ^+^ CD8^+^ T cell responses with cell lines generated with the Spike-derived peptide pool and restimulated with either the Spike-derived peptide pool (**H**) or with the HLA-A*03:01-restricted KCY peptide (**I**) before vaccination (V0) and after the first (V1) and second (V2) vaccine doses. (**J**) Paired comparison of KCY- and influenza NP_265_^−^ specific responses showing IFNγ production by CD8^+^ T cells. Bars represent mean ± SD; symbols denote individual donors. V0, pre-vaccination; V1, post-dose 1; V2, post-dose 2; V5, > 400 days post-dose 3. *p < 0.05; **p < 0.01.

To examine the population of Spike-specific CD8^+^ T cells present before and after vaccination, we considered the reported dominant HLA-A*03:01-restricted Spike-derived epitope _378_KCYGVSPTK_386_, hereafter named KCY^20,38^. It has been shown that KCY-specific memory T cells increase upon viral exposure or vaccination^20^. Therefore, we performed *ex vivo* tetramer-associated magnetic enrichment (TAME) to analyze the phenotype of the KCY^+^ CD8^+^ T cells in samples collected before and after vaccination (Figure 2A). Surprisingly, even prior to vaccination (V0) a high frequency of KCY^+^ T cells was present in all samples (Figure 2A). The frequency of KCY^+^ T cells increased after vaccination (Figure 2B–D). Interestingly, after vaccination, a distinct population of KCY^+^ T cells with high mean fluorescence intensity (MFI) emerged (Figure 2A, **D**). The high MFI KCY^+^ T cell population expanded significantly after vaccination compared to baseline (V0 mean: 1.49 ± 0.89; V1 mean: 7.49 ± 2.01; V2 mean: 5.53 ± 0.65) (Figure 2D). In comparison, TAME with the HLA-A*02:01- restricted S_269–277_ peptide^39–41^ did not show tetramer^+^ T cells before vaccination, and only a high MFI population of tetramer^+^ T cells was observed after vaccination(**Supplementary Figure 6A**). We also confirmed that the large population of KCY tetramer^+^ T cells observed *ex vivo* was specific to the KCY peptide and not binding to an HLA-A*03:01-restricted influenza-derived peptide that we characterized previously, called Flu-NP_265_^42^ (Figure 2E).

Given the large frequency of KCY^+^ T cells *ex vivo*, we wondered if this population would be expanded after activation *in vitro*. To test this, we generated T cell lines using PBMCs cultured with either the KCY peptide or an HLA-A*03:01-restricted influenza-derived peptide, called Flu-NP_265_ that we previously characterized^42^, to be tested in the same samples as the KCY peptide. The KCY T cell lines showed specificity for the KCY peptide only, and the Flu-NP_265_ T cell lines were specific only to the Flu-NP_265_ peptide (**Supplementary Figure 5B**). The frequency of KCY^+^ CD8^+^ T cells *in vitro* was low (average of 0.71 ± 0.39 %, n = 3) (**Supplementary Figure 5B**), while it was 8-times higher for the Flu-NP_265_^+^ T cells (average of 5.71 ± 1.67 %, n = 3) (**Supplementary Figure 5B**). This is in contrast with the high frequency observed *ex vivo* (Figure 2A); however, the *ex vivo* data were obtained after TAME. Therefore, we compared tetramer staining in *ex vivo* and *in vitro* samples, without tetramer magnetic enrichment. We observed that there was a 16- and 44-fold lower frequency of KCY^+^ T cells *in vitro* compared to *ex vivo* in vacSG82-V2 and vacSG88-V2 samples, respectively (**Supplementary Figure 5C**). In addition, while the majority of *ex vivo* KCY^+^ T cells had low MFI, the *in vitro* cells were largely high MFI (**Supplementary Figure 5C**).

Overall, even prior to vaccination, an unusually large number of low avidity KCY-specific T cell populations was present in HLA-A*03:01^+^ samples *ex vivo* that were not expanded upon KCY presentation. In addition, vaccination led to an increase in high avidity KCY^+^ T cell population, although these cells remained present at low frequencies relative to the low avidity population.

### KCY^+^ T cells mostly exhibit a naïve phenotype

We next assessed the phenotype of KCY^+^ T cells *ex vivo* to determine if there was a difference between the high and the low avidity population (Figure 2F–G, **Supplementary Figure 6A**). The majority of the low MFI KCY^+^ T cell population exhibited a naïve phenotype (T_N_: CCR7^+^/CD45RA^+^) independent of the vaccine status of the donors (average of 69.95, 56.35 and 63.9 % for V0, V1 and V2, respectively), and some stem cell memory T cells (T_SCM_: CCR7^+^/CD45RA^+^/CD95^+^) present especially in one donor (V1) (Figure 2F, **Supplementary Figure 6A**). The low MFI effector memory T cell (T_EM_: CCR7^−^/CD45RA^−^) frequency was ~16%, and the terminally differentiated effector T cell (T_EMRA_: CCR7^−^/CD45RA^+^) frequency was around ~10% independent of vaccination status.

The comparison of high and low MFI tetramer^+^ CD8^+^ T cell phenotype showed that while the proportion of low MFI naïve T cells remains the same before and after vaccination (Figure 2F, **Supplementary Figure 6A**), the high MFI naïve T cell proportion decreased after vaccination while high MFI effector and central memory T cell proportion increased (Figure 2G, **Supplementary Figure 6A**). Strikingly, the phenotype of the KCY^+^ T cells observed in HLA-A*03:01^+^ samples was different from the one observed for the S_269_ peptide in HLA-A*02:01^+^ samples (**Supplementary Figure 6B**). We did not observe any S_269_^+^ T cells in V0 samples, and S_269_^+^ T cells were only observed in 1 out of 3 donors after V1 with a T_CM_ and naïve phenotype (**Supplementary Figure 6B-C**). In V2 samples T_EM,_ but no T_EMRA_ cells, were observed. This is in contrast with the presence of both KCY^+^ T_EM_ and T_EMRA_ cells, even prior to vaccination (Figure 2F–G, **Supplementary Figure 6C**). In two samples collected > 400 days after the 3^rd^ vaccine dose (vacSG64-V5 and vacSG86-V5), we could observe 36% and 8.85% of KCY^+^ T_EMRA_ cells, respectively, and a large population of naïve cells (32% and 79.7%, respectively) (**Supplementary Figure 6D**).

Overall, despite the naïve phenotype of the large proportion of low avidity KCY-specific T cells, the cells are peptide specific. In addition, the high avidity effector memory cells are present before vaccination, increased after vaccination and persist over time. Nevertheless, the vast majority of KCY-specific T cells remain low avidity, naïve, and in high numbers.

### Spike-specific T cell activation in HLA-A*03:01^+^ donors is relatively weak

To understand whether the T cell response was likely responsible for the observed vaccine reactogenicity in HLA-A*03:01^+^ donors, T cell lines were generated with Spike-derived peptide pools covering the whole length of the Spike protein and restimulated with either the peptide pools or the KCY peptide. The response before vaccination (V0) against the Spike-derived peptide pools was low for both CD8^+^ and CD4^+^ T cells (Figure 2H, **Supplementary Figures 7 – 10**). A trend upward of IFNγ producing CD8^+^ T cells was observed after vaccination (V1; mean ± SD; 0.09 ± 0.08 %, and V2; 0.39 ± 0.48 %) (Figure 2H), but not for CD4^+^ T cells (V1: 0.02 ± 0.04 %, and V2: 0.02 ± 0.07 %) (**Supplementary Figures 10A**).

We also assessed the KCY peptide specific response. We observed limited IFNγ production in 50% of the samples before vaccination (n=4/8, 0.05 ± 0.06 %), and a larger IFNγ production in 85% (n=6/7, average of 0.16 ± 0.19 %) and 57% (n=4/7, average of 0.15 ± 0.19 %) after the first and second vaccine dose, respectively (Figure 2I, **Supplementary Figures 7–8**). Despite the increase of IFNγproducing KCY-specific CD8^+^ T cells after vaccination, the response was overall weak compared with other well characterized Spike-derived epitopes such as HLA-A*02:01-restricted S_269–277_ (average of 5.85% IFNγ^+^ CD8^+^ T cells^39^ or HLA-B*15:01-restricted S_919–927_ (average of 0.36% IFNγ^+^ CD8^+^ T cells in pre-pandemic samples^23^). In addition, we previously characterized an HLA-A*03:01-restricted influenza-derived peptide, called Flu-NP_265_^42^ that was also tested here in the same samples side by side with the KCY peptide. The response to the Flu-NP_265_ peptide was substantially stronger than that to the KCY peptide, with IFNγ production being ~13-fold higher (Flu-NP_265_: 3.32 ± 2.36 %; KCY: 0.27 ± 0.19 %) (Figure 2J, **Supplementary Figure 8**, **10D**), TNF production ~25-fold higher (Flu-NP_265_: 3.31 ± 2.28 %; KCY: 0.13 ± 0.06 %), and CD107a ~10-fold higher than KCY (Flu-NP_265_: 3.32 ± 2.36 %; KCY: 0.34 ± 0.33 %) (**Supplementary Figure 10E**).

The response towards the Flu-NP_265_ peptide demonstrated that HLA-A*03:01^+^ CD8^+^ T cells can produce high level of cytokines. However, the CD8^+^ T cell response towards the Spike-derived peptides, even for the dominant KCY peptide, was overall weak even after vaccination, and therefore unlikely to underpin the vaccine side effect correlation observed in HLA-A*03:01^+^ individuals.

### Full-length Spike protein stimulated high expression of IL-6 and IL-8 by monocytes

Despite the high number of KCY^+^ T cells present (Figure 2A), the overall T cell response did not show the high levels of cytokine production that could explain vaccine reactogenicity associated with HLA-A*03:01 carriage (Figure 2H–I). Therefore, we asked whether other immune cells could lead to inflammation that would underpin vaccine side effects. To address this, we used the different vaccine components to stimulate PBMCs; the empty lipid nanoparticle (LNP) using the Pfizer vaccine formula^43^ the soluble full-length Spike protein (HexaPro)^44^, or the Spike-derived peptide pools (S1 and S2), as well as positive and negative controls.

**Figure 3:**
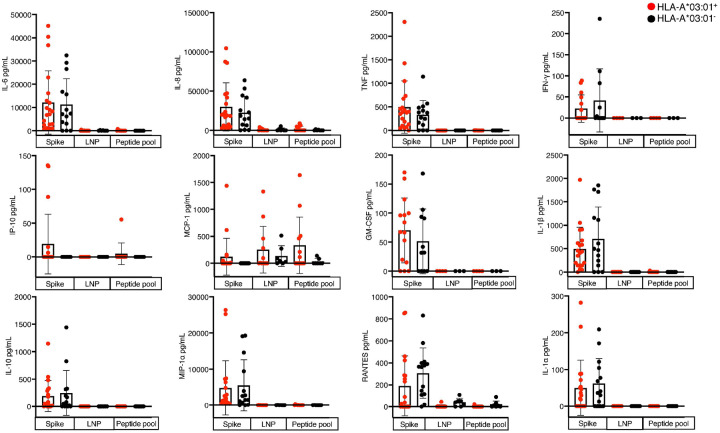
Spike stimulation activates a large IL-6 and IL-8 production Cytokine levels measured in PBMCs from HLA-A*03:01^+^ (n = 19, red dots) and HLA-A*03:01^−^ (n = 16, black dots) samples after stimulation with full-length soluble Spike protein, Spike peptide pool, or lipid nanoparticle (LNP) control. Only full-length Spike induced broad cytokine responses, including IL-6, IL-8, IP-10, GM-CSF, TNF, MCP-1, IL-1β, IL-10, MIP-1α, RANTES, IL-1α and IFNγ. Bars represent mean ± SD; symbols denote individual donors.

Among the 20 HLA-A*03:01^+^ and 14 HLA-A*03:01^−^ PBMC samples tested (**Supplementary Table 20**), detectable cytokine production was only observed in samples stimulated with the full-length soluble Spike protein (Figure 3, **Supplementary Figure 11**). Neither the Spike-derived peptide pools nor the LNPs induced measurable cytokine responses in either group, except for Monocyte Chemoattractant Protein-1 (MCP-1) (Figure 3). Although MCP-1 levels showed a modest increase in HLA-A*03:01^+^ samples following Spike stimulation (120.7 ± 340.9 pg/mL) compared with HLA-A*03:01^−^ samples (0.0 ± 0.0 pg/mL), the response remained within the expected baseline variation observed in healthy individuals (~250 pg/mL), indicating no biologically meaningful induction of MCP-1 by Spike stimulation. Similar response was observed for eLNP and Spike Pool stimulations (253.4 ± 432.1 pg/mL and 333.3 ± 523.5 pg/mL, respectively). Cytokines induced by whole Spike stimulation, included Interleukin (IL)-6, IL-8, IL-1a, IL-1b, IL-10, macrophage inflammatory protein-1a (MIP1a), granulocyte-macrophage colony-stimulating factor (GM-CSF), RANTES, MCP-1, tumor necrosis factor (TNF), inducible protein 10 kDa (IP-10), and Interferon-γ (IFNγ) (Figure 3, **Supplementary Figure 11**). The increase was observed in both HLA-A*03:01^+^ and HLA-A*03:01^−^ samples, and no significant difference in the level of cytokine was observed between the groups. However, IL-6 and IL-8 were modestly elevated in HLA-A*03:01^+^ samples after Spike stimulation (12,186 ± 13,659 pg/mL and 29,875 ± 30,682 pg/mL, respectively), representing ~1.1- and ~1.3-fold increase compared with IL-6 (11,234 ± 11,177 pg/mL) and IL-8 (22,436 ± 20,784 pg/mL) levels in HLA-A*03:01^−^ samples (Figure 3). IL-6 and IL-8 cytokines were also expressed at the highest concentration compared to other cytokines.

**Figure 4:**
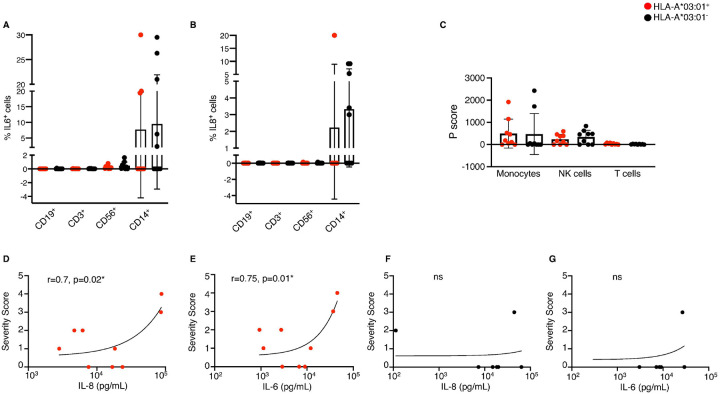
IL-6 and IL-8 cytokines are mainly produced by monocytes and correlates with vaccine side-effect severity for HLA-A*03:01^+^ donors (A-B) Intracellular cytokine staining (ICS) of Spike-stimulated PBMCs showing IL-6^+^ (**A**) and IL-8^+^ (**B**) cells among CD3^+^ T cells, CD19^+^ B cells, CD56^+^ NK cells, and CD14^+^ monocytes from HLA-A*03:01^+^ (red dots) and HLA-A*03:01^−^ samples (black dots). (**C**) Phagocytic score (P score) representing the uptake of Spike-coated microbeads by monocytes, NK cells and T cells in HLA-A*03:01^+^ (red dots) and HLA-A*03:01^−^ samples (black dots). Bars represent mean ± SD; symbols denote individual donors. (**D-G**) Correlation of IL-8 (**D**, **F**) and IL-6 (**E**, **G**) concentrations with self-reported vaccine side-effect severity scores from HLA-A*03:01^+^ (red dots) and HLA-A*03:01^−^ donors (black dots).

We next asked which cell subset was responsible for the production of IL-6 and IL-8 upon Spike stimulation. To address this, cytokine production was assessed in Spike-stimulated PBMCs using flow cytometry using multiple cell surface markers. IL-6 was produced predominantly by monocytes (CD14^+^) despite the low number of CD14^+^ cells in blood (**Supplementary Figure 12A**-**B**), and at lower levels by Natural Killer (NK) cells (CD56^+^), suggesting a primary role of the innate response in vaccine reactogenicity (Figure 4A). In HLA-A*03:01^+^ samples, IL-6^+^ NK cells averaged 0.23 ± 0.23 % (n = 9/9 positive) and monocytes 7.7 ± 11.9 % (n = 3/9), whereas in HLA-A*03:01^−^ samples comparable NK responses (0.45 ± 0.56 %, n = 7/9) but broader monocyte positivity (9.5 ± 12.4 %, n = 5/9) were observed (Figure 4A). In contrast, IL-6^+^ T and B cells were rare, with only traceable responses (< 0.03 %, n ≤ 6/9 donors). IL-8 responses were even more restricted. Only a minority of monocyte-positive donors showed detectable IL-8, averaging 2.2 ± 6.7 % (n = 1/9) in HLA-A*03:01^+^ and 3.3 ± 3.8 % (n = 5/9) in HLA-A*03:01^−^ samples. Other subsets (NK, T, and B cells) produced negligible IL-8 (< 0.02 %, n ≤ 5/9 donors) (Figure 4B). Compared to the IL-6 and IL-8 levels secreted (Figure 3A), the levels of IL-6^+^ and IL-8^+^ cells detected were at low frequencies in PBMCs, therefore, we examined the Spike uptake capacity by the PBMCs. Phagocytic scores (engulfment of Spike-coated microbeads; Figure 4C, **Supplementary Figure 12C**) confirmed that Spike uptake was mainly driven by monocytes and at lower level by NK cells.

Overall, the high level of IL-6 and IL-8 production was observed only in the presence of the full Spike protein and was primarily produced by monocytes.

### Expression of IL-6 and IL-8 correlates with side effect severity in HLA-A*03:01 donors

While our results suggest a role for IL-6 and IL-8 in vaccine reactogenicity, the differences observed between grouped HLA-A*03:01^+^ and HLA-A*03:01^−^ donor samples were not sufficient to explain the observed differences in response to vaccination. However, as with most complex phenotypes, the association of HLA-A*03:01^+^with vaccine reactogenicity is incompletely penetrant. While more individuals carrying this allele report SSE with vaccination, there was a range of severity reported among our PBMC donors; thus, we sought to determine whether levels of IL-6 and IL-8 were correlated with reported side effect severity in these donors. Strikingly, in HLA-A*03:01^+^ samples, both IL-8 and IL-6 levels showed strong and significant positive correlations with severity score (IL-8: r = 0.70, p = 0.02; IL-6: r = 0.75, p = 0.01; Figure 4D–E), indicating that higher cytokine production was associated with increased vaccine side effect severity. In contrast, no significant correlation was observed in HLA-A*03:01^−^ individuals (Figure 4F–G). To ensure that the correlation observed in HLA-A*03:01^+^ samples was due to the transient presence of Spike protein, we checked the baseline levels of IL-6 and IL-8 cytokines in serum of HLA-A*03:01^+^ collected prior to vaccination, or two-weeks post first and second dose of vaccine, alongside TNF and IFNγ as control (**Supplementary Figure 13**). Overall, the level of cytokines was low (< 100 pg/mL) or moderate, and no significant increase was observed before or after vaccination. This demonstrates that the cytokine production upon Spike presentation is likely transient.

Together, these data establish that HLA-A*03:01^+^ samples display a distinct pro-inflammatory signature, with IL-6 and IL-8 production strongly linked to vaccine side-effect severity. The early and transient nature of this cytokine production primarily by monocytes and NK cells strongly suggests an innate immune response underlying HLA-A*03:01 vaccine reactogenicity.

### HLA-A*03:01 is an eQTL for IRF4 driving monocyte differentiation

Finally, to better understand the relationship between HLA-A*03:01 carriage and the observed role of innate immune cells in vaccine reactogenicity, we examined patterns of differential gene expression driven by HLA-A*03:01. Limiting our analysis to genes on chromosome 6 (723 tests), differential gene analysis for 21 donors showed that *IRF4*, which is involved in monocyte differentiation to dendritic cells (DCs) and homing to lymph nodes^45^, is significantly upregulated in HLA-A*03:01 donors (log fold 1.863, p-value = 9.56 × 10^−6^, p_adj_ = 0.0069) (**Supplementary Table 22**). Thus, it appears that the HLA-A*03:01 association with SARS-CoV-2 vaccine reactogenicity may be in part due to its role as an eQTL, resulting in increased differentiation of monocytes to DCs, increasing antigen-presenting cells and homing them to the lymph nodes.

## Discussion

Vaccination has been a crucial public health intervention for decades, significantly contributing to the prevention and control of infectious diseases worldwide. However, concerns about vaccine safety and efficacy, and negative public perceptions regarding side effects, have emerged as a significant challenge in maintaining high vaccination coverage. Because the binding between HLA and peptide antigen is highly specific and a fundamental component in initiating the adaptive immune system, understanding the role of *HLA* variation in vaccine response can be crucial in determining factors that underlie the effectiveness of vaccination. Variation in HLA has previously been reported as associated with SARS-CoV-2 vaccine reactogenicity^30,31,37^. Here, in a much larger cohort than previously examined, we sought to refine and understand the relationship between variation at all *HLA* loci and reports of side effects associated with the vaccine.

We leveraged a large, registry-based cohort of more than 50,000 individuals to provide the necessary statistical power and diversity to reliably identify genetic factors that influence individual susceptibility to systemic reactions following vaccination. Variation in the HLA region has previously been associated with interindividual differences in humoral immune responses after vaccination. For example, *HLA* variation has been linked to either increased or decreased immune responses to influenza^46^, measles^47^, rubella^48^, and hepatitis B vaccination^49^, respectively. In COVID-19 vaccination, previous reports have shown a suggested association of HLA variation with specific systemic mild side effects such as fever and fatigue, including HLA-A*03:01^30^.

Here, we showed significant associations of HLA-A*03:01 with increased side effects such as fever, chills, and muscle pain, particularly with higher effect sizes in individuals who received the Pfizer vaccine. Evidence from this and prior studies^19,50^ demonstrates that HLA-A*03:01 is also significantly associated with high serum levels of anti-SARS-CoV-2-Spike antibodies. This supports the notion that this allomorph promotes an especially robust immune response to vaccination for SARS-CoV-2, including both cell-mediated and humoral immunity. Additionally, we demonstrated that a negative correlation of HLA-A*03:01 with BTI is likely mediated by the systemic inflammatory response that causes vaccination adverse effects. To fully contextualize the findings, we compared responses to COVID-19 vaccines with those of influenza vaccine. Our finding of no significant association of HLA-A*03:01 with side effects from influenza vaccine (or any other *HLA* allele) demonstrates that the observed HLA-A*03:01 associated vaccine reactogenicity is specific to the COVID-19 vaccine.

Owing to the crucial role of HLA class I molecules in antigen presentation, the role of CD8^+^ T cells in HLA-A*03:01-mediated vaccine reactogenicity presented a clear initial line of inquiry into the mechanisms underlying reported side effects. We observed a high frequency of CD8^+^ T cells able to recognize the immunodominant Spike-derived peptide KCY specifically^20^, even prior to vaccination or infection. Interestingly, both prior to and subsequent to vaccination, the majority of the CD8^+^ T cells exhibited low MFI (Mean Florescence Intensity), suggesting that the cells are low avidity; in addition, a large proportion of those peptide-specific cells had a naïve phenotype. This contrasts with our previous study on a dominant Influenza-derived peptide, Flu-NP_265_^42^ for which we observed low and high MFI cells *ex vivo*, consistent with the fact that prior viral exposure and/or vaccination leads to the presence of high MFI T cells. Post-COVID-19 vaccination we did observe the expansion of high MFI KCY-specific CD8^+^ T cells and an increased proportion of memory phenotype. However, in contrast with HLA-A*02:01^+^ samples, even after multiple doses of the COVID-19 vaccine, the frequency of KCY^+^ naïve CD8^+^ T cells remained high, likely reflecting the large proportion of naïve T cells able to specifically bind the KCY peptide independent of vaccine status. Most strikingly, even post-vaccination, the T cell response to KCY was muted relative to responses to Influenza-derived peptide, such that it is unlikely that the observed COVID-19 vaccine reactogenicity can be attributed to a robust T cell activation.

Monocytes thus emerged as a central population of interest: they were the predominant source of IL-6 and IL-8 following Spike stimulation, driving the pro-inflammatory response that correlated with side-effect severity in HLA-A*03:01^+^ donors. Strikingly, this cytokine induction was accompanied by a reduction of the already low frequency of CD14^+^ monocytes in blood, suggesting a shift in lineage fate rather than simple activation, consistent with our RNA-sequencing results. Our findings reveal a distinct gene expression signature associated with HLA-A*03:01, suggesting upregulation of the monocyte to DC differentiation pathway. Increased *IRF4* expression in HLA-A*03:01^+^ individuals suggests increased DC priming and more efficient migration to lymph nodes^45^. Given that CD14 downregulation is a hallmark of monocyte-to-dendritic cell differentiation, our results suggest that HLA-A*03:01^+^ individuals likely have higher frequencies of dendritic cells, highly efficient antigen presenters, homed to the lymph node than HLA-A*03:01^−^ individuals.

Despite what appears to be the central role of the monocyte-DC lineage in cytokine production associated with vaccine reactogenicity, the unusually high numbers of low-avidity, naïve, Spike-specific CD8^+^ T cells observed in HLA-A*03:01^+^ donors likely provide an inflammatory milieu in the lymph node after vaccination. While these T cells do not appear to become activated themselves, their binding to peptide-HLA presented by DCs at high frequency may constitute a signal of immune activity for DCs^51^; this likely contributes to activation of these DCs, resulting in increased production of IL-6 and other cytokines^52,53^. Thus, we postulate that in HLA-A*03:01^+^ individuals, a high number of low-avidity, naïve T cells is available to bind to already primed DCs stimulated by Spike, resulting in an amplified immune response after COVID-19 vaccination.

This study is intrinsically constrained by its dependence on self-reported data to assess transient mild vaccine side effects in both the discovery and replication cohorts, which potentially can lead to some imprecision in association results. Additionally, sample size limitations restrict some significant findings to individuals who self-identify as White. Likewise, our cohort was predominantly female (78.4%), which may limit the generalizability to broader populations. Moreover, the low number of monocyte cells in the blood limited the ability to show significant differences, in addition to the incomplete penetrance of the observed genetic effect, which is typical for complex traits. The transient features of SSE also constrained these observations.

Despite these limitations, our findings regarding the role of *HLA*-mediated COVID-19 vaccine reactogenicity and the associated evidence for protection from subsequent infection provide important and novel insights regarding these responses, which may inform efforts toward improved vaccine efficacy and increased public participation in vaccination programs.

## Methods

### Discovery cohort

We recruited our study population via email to all potential volunteer bone marrow donors registered in the NMDP database with available email addresses and high-resolution HLA genotyping information available. The email contained a custom link directing them to a consent page for a health history survey. Subject recruitment, consent process, and survey administration were conducted using both email outreach and a web interface to ensure effective data collection. Upon consenting to provide responses and allow linking with their HLA genotype data, participants spent ten to fifteen minutes completing a detailed survey to gather baseline information and health history.

As of January 19th, 2024, a total of 80,016 eligible donors completed the survey. Among these respondents, 667 individuals (0.83%) were excluded for filling out the survey multiple times, while 1,301 participants were removed due to incomplete HLA variation data. After excluding these cases and individuals that were also participated in the study that formed our replication cohort, below (N = 836), there remained a total of 77,212 participants in the study. Of these individuals, 56,938 people who self-identified as White, Hispanic, African American, or Asian Pacific Islanders, had completed their initial series of vaccinations. We excluded participants who identified as multiple ancestry, unknown ancestry or Native American due to small sample sizes.

### Replication cohort

Our replication cohort consisted of participants who participated in a prior study with NMDP tracking experiences with COVID-19 through a mobile app, described in detail in Augusto et al. 2023^23^. Once enrolled, the participants are asked to complete an initial 10 to 15-minute survey about baseline demographics, their health history, and daily habits. Follow-up daily questions specific to vaccine side effects are delivered by push notification or text message on an ongoing basis and require 5 to 15-minute per week. All the participants provided written informed consent agreeing to the research and publication of research results.

We restricted our analysis to individuals who had self-identified as ‘White’ (which we use as a proxy for European ancestry) due to insufficient numbers for analysis in the other groups, allowing an analysis of 10,595 individuals reporting vaccination for SARS-CoV-2. Of those, 4,575 individuals completed their initial series of vaccinations. Symptoms are self-reported at the baseline and in daily surveys. Within the baseline survey, the respondents were asked to report whether they had any of a list of symptoms (**Supplementary Table 23**) for 3 days or longer at any time after their complete dose of vaccination.

### Serum antibody levels in vaccinated subjects

The population examined consisted of 156 healthcare workers from “Evangelismos” General Hospital in Athens, Greece, including doctors, nurses, pharmacists, biologists, dentists, technicians and administrative staff. Enrollment was open to all hospital personnel scheduled for vaccination and not restricted by any pre-specified criteria. All individuals received two doses of the mRNA Pfizer-BioNTech vaccine. Data on prior SARS-CoV-2 infection and symptoms experienced after each dose were collected for all participants. Antibody concentrations were assessed at two time points: 21 ± 1 days after the first dose and 24 ± 2 days after the second dose. Levels of circulating SARS-CoV-2 anti-Spike IgG (S) and anti-nucleocapsid IgG (N) antibodies were quantified using the Abbott Diagnostics SARS-CoV-2 IgG chemiluminescent microparticle immunoassay (Abbott Diagnostics, Abbott Park, Illinois) on an Abbott Diagnostics Architect i2000 SR and an Alinityi Analyzer, according to the manufacturer’s instructions. Results were expressed in AU/mL and were interpreted as positive if ≥ 50 AU/mL^54^. Informed consent was obtained from all participants, and the study was approved by the Institutional Review Board of “Evangelismos” Hospital (PN 9/21-01-21). High resolution HLA class I and II genotyping was performed as described^55^.

### Peripheral blood mononuclear cells (PBMCs)

HLA-A*03:01^+^ and HLA-A*03:01^−^ donors vaccinated with either the Comirnaty BNT162b2 COVID-19 mRNA vaccine (Pfizer) or the Oxford–AstraZeneca COVID-19 vaccine (AstraZeneca), and most of them naïve for SARS-CoV-2 infection, were recruited (**Supplementary Table 20**). PBMCs were separated from whole blood or buffy coats using density-gradient centrifugation. PBMCs were used fresh or were cryogenically stored until use. All individuals consented to research and publication of research results and had been previously HLA genotyped. Ethics approval to undertake the research was obtained from the La Trobe University Human Research Ethics Committee (HEC21097). The HLA genotyping was performed by AlloSeq Tx17 (CareDx Pty) using AllType NGS high-resolution genotyping on the IonTorrent NGS platform or by the Department of Clinical Immunology and PathWest at Fiona Stanley Hospital, Murdoch, Australia.

### Tetramer-associated magnetic enrichment (TAME)

Peptide-loaded HLA-A*03:01 tetramers were generated using Streptavidin conjugated to phycoerythrin (PE). Tetramer-stained cells were enriched using anti-PE antibody-coated immunomagnetic beads on LS columns (Miltenyi Biotech) according to manufacturer instructions. After enrichment, cells were stained with an antibody panel including anti-CD3-BV480 (dilution 1:100), anti-CD8-PerCP-Cy5.5 (1:50), anti-CD4-FITC (1:100), anti-CD14-APCH7 (1:200), anti-CD19-APCH7 (1:100), anti-CD45RA-BUV395 (1:100), anti-CD27-APC (1:100), anti-CCR7-PE-Cy7 (1:50), anti-CD95-BV421 (1:50), anti-PD1-BV605 (1:100), anti-CXCR5-BV650 (1:100) (all BD Biosciences) and Live/Dead Fixable Near-IR Dead Cell Stain (1:1,000) (Life Technologies). Cells were resuspended in MACS buffer (PBS, 0.5% BSA, 2 mM EDTA) and were analysed using the BD FACSymphony A3 system.

For the tetramer staining experiments, the TAME cells were stained for 1 hour at room temperature with the APC-conjugated Flu-NP_265_ HLA-A*03:01-restricted peptide tetramer, followed by surface staining using the same antibody panel as above, excluding anti-CD27-APC. Gating strategy shown on **Supplementary Figure 14**.

### Generation of peptide-specific CD8^+^ T cell lines

CD8^+^ T cell lines were generated as previously described^56,57^. In brief, PBMCs were incubated with 1 μM of individual SARS-CoV-2 Spike-derived peptide or 10μg/mL of Spike-derived peptide Pool 1 (25 μg/peptide, 15mers, 1 – 126) and Pool 2 (25 μg/peptide, 15mers, 127 – 253) (Mimotopes B#33200); and cultured for 10 – 14 days in RPMI-1640 supplemented with 2 mM MEM non-essential amino acid solution (Sigma-Aldrich), 100 mM HEPES (Sigma-Aldrich), 2 mM l-glutamine (Sigma-Aldrich), penicillin–streptomycin (Life Technologies), 50 mM 2-ME (Sigma-Aldrich) and 10% heat-inactivated fetal bovine serum (Bovogen). The cultures were supplemented with 10 IU IL-2 2 – 3 times weekly. CD8^+^ T cell lines were used fresh for subsequent analysis.

For the tetramer staining experiments 0.5 × 10^6^ cells from the CD8^+^ T cell lines were stained with a PE-conjugated tetramer (HLA-A*03:01-KCY) or double-stained with two tetramers (PE-conjugated KCY and APC-conjugated Flu-NP_265_ HLA-A*03:01-restricted peptide tetramer) for 1 h at room temperature. Cells were washed and surface-stained with anti-CD3-BV480 (dilution 1:100), anti-CD8-PerCP-Cy5.5 (1:50), anti-CD4-BV650 or -FITC (1:100), anti-CD14-APCH7 (1:200) and anti-CD19-APCH7 (1:100) antibodies (all BD Biosciences) and Live/Dead Fixable Near-IR Dead Cell Stain (Life Technologies). Cells were analysed using the BD FACSymphony A3 system.

### Intracellular cytokine assay

CD8^+^ T cell lines were stimulated with 1 μM of individual peptide or 2μg/mL of the SARS-CoV-2 Spike-derived peptide Pool 1 (25 μg/peptide, 15mers, 1 – 126) and Pool 2 (25 μg/peptide, 15mers, 127 – 253) (Mimotopes B#33200) and were incubated for 4 – 5 hour in the presence of GolgiPlug, GolgiStop and anti-CD107a-FITC (dilution 1:100) (all BD Biosciences). After stimulation, cells were surface stained for 30 min with anti-CD3-BV480 (1:100), anti-CD8-PerCP-Cy5.5 (1:50) and anti-CD4-BV650 (1:100) antibodies (all BD Biosciences) and Live/Dead Fixable Near-IR Dead Cell Stain (Life Technologies). Cells were fixed and permeabilized using BD Cytofix/Cytoperm solution (BD Biosciences) and then intracellularly stained with anti-IFN-γ-BV421 (1:100), anti-TNF-PE-Cy7 (1:100), anti-IL2-PE (1:100) and anti-MIP-1β-APC (1:100) antibodies (all BD Biosciences) for a further 30 min. Cells were acquired on the BD FACSymphony A3 system using the FACSDiva software (v.9.0.). Post-acquisition analysis was performed using FlowJo software (v.10). Cytokine detection levels identified in the no-peptide control condition were subtracted from the corresponding test conditions in all summary graphs to account for non-specific, spontaneous cytokine production. Gating strategy shown on **Supplementary Figure 14**.

### PBMCs short-term stimulation

1 × 10^6 PBMCs were stimulated with either 15 μg/mL of empty Pfizer-BioNTech lipid nanoparticle (LNP)^43^ or 15 μg/mL custom made SARS-CoV-2 Spike protein (Wuhan strain); or 7.5 μg/mL of the SARS-CoV-2 Spike-derived peptide Pool 1 (25 μg/peptide, 15mers, 1 – 126) and 7.5 μg/mL Pool 2 (25 μg/peptide, 15mers, 127 – 253) (MIMOTOPES B#33200); or Cell Stimulation Cocktail (500X) (eBioscience^™^); or nothing (Negative Control) for 15 hours in RPMI-1640 supplemented with 2 mM MEM non-essential amino acid solution (Sigma-Aldrich), 100 mM HEPES (Sigma-Aldrich), 2 mM l-glutamine (Sigma-Aldrich), penicillin–streptomycin (Life Technologies), 50 mM 2-ME (Sigma-Aldrich) and 10% heat-inactivated fetal bovine serum (Bovogen). Following the 15-hour stimulation, the cells were restimulated for a further 2-hour using the same conditions and the supernatant was collected for the BD Cytometric Bead Array (CBA). The same conditions were also used in the presence of GolgiPlug and GolgiStop (BD Biosciences). Following the 15-hour stimulation and the 2-hour restimulation, the cells were surface-stained for 30 minutes with anti-CD3-BV480 (1:100), anti-CD14-PerCP-Cy5.5 (1:100), anti-CD16-BV421 (1:50), anti-CD19-APC (1:50), anti-CD56-PECy7 (1:50) antibodies (all BD Biosciences) and Live/Dead Fixable Near-IR Dead Cell Stain (Life Technologies). Cells were fixed and permeabilized using BD Cytofix/Cytoperm solution (BD Biosciences) and then intracellularly stained with anti-IL6-FITC (1:50) and anti-IL8-PE (1:50) antibodies (both BD Biosciences) for a further 30 minutes. Cells were acquired on the BD FACSymphony A3 system using the FACSDiva software (v.9.0.). Gating strategy shown on **Supplementary Figure 15**.

### BD Cytometric Bead Array (CBA)

Following the PBMC short-term stimulation, the supernatant level of Interleukin (IL)-1α, IL-1β, IL-2, IL-4, IL-5, IL-6, IL-7, IL-8, IL-9, IL-10, IL-12p70, IL-13, Interferon (IFN)-α, IFN-γ, IFN-γ inducible protein 10 kDa (IP-10), granulocyte-macrophage colony-stimulating factor (GM-CSF), Lymphotoxin-alpha (LT-α), Eotaxin, Monocyte Chemoattractant Protein-1 (MCP-1), macrophage inflammatory protein-1 alpha (MIP-1α), RANTES, tumor necrosis factor (TNF) and were measured using the BD Cytometric Bead Array (CBA, BD Biosciences) following the manufacturer’s instructions. Samples were acquired in a BD FACSymphony A3 system using the FACSDiva software (v.9.0.). The analysis was performed by using the FCAP Array Software v3.0. Cytokine detection levels identified in the Negative Control condition were subtracted from the corresponding test conditions in all summary graphs to account for non-specific, spontaneous cytokine production.

### SARS-CoV-2 specific phagocytosis assays

After PBMCs stimulation with full length Spike protein for 15-hour, PBMCs (1 × 10^5^ cells in 50μL) were added onto 60 μL of the donor plasma opsonised microbeads (10μL plasma and 50μL microbeads) in a 1.5mL Eppendorf tube, mixed by gentle tapping, adjusted to 600 μL using RPMI 1640 containing 0.1% Human serum and 0.1 M HEPES pH 7.4 and transferred into 37°C, 5 % CO_2_ incubator. After 2-hour of incubation, cells were washed once with 1mL of cold PBS containing 0.5 % FBS and 0.005 % sodium azide and gentle centrifugation at 335 × g for 5 minutes at 4°C, fixed in 400 μL of 1 % paraformaldehyde, and kept at 4°C in the dark until the acquisition of data using BD FACSCaliburTM Flow cytometer. A total of 2 ×10^4^ events per tube were acquired from each donor conditions. Relevant assay controls included the acquisition of 2 ×10^4^ events per tube from cells incubated with no beads, Spike-coated nonopsonized beads. The proportions of cells that phagocytosed the beads (% of cells that took up the beads) and their fluorescent intensities (amounts of beads taken up per cell) were analyzed using BD FlowJo version 10.5.0 software. Phagocytic scores (p-score) were then calculated based on the proportion of cells that took up the opsonized beads denoting the number of positive cells and mean fluorescence intensity (MFI) representing the average bead uptake by the positive cells as described. A positive p-score was defined as three standard deviations above the background mean phagocytic score of healthy donors as described previously^58^.

In selected experiments, the intracellular uptake of the opsonized microbeads by effector cells was confirmed by confocal microscopy as described^58^. In brief, PBMC after (1 × 10^4^ cells) after phagocytosis assay were washed twice with cold PBS containing 0.5% FBS and 0.005 % of sodium azide, fixed with 1% paraformaldehyde for 5 minutes at room temperature, and rinsed twice with PBS. The fixed cells were blocked with 1% BSA in PBS, incubated with 1:1000 dilution of Alexa-555-conjugated Phalloidin (Sigma, USA) for 30 minutes at room temperature, mounted in DAPI nuclear stain-containing media (Molecular Probes, USA), and imaged using ZEISS LSM 880 confocal microscope (Carl Zeiss AG, Germany), using 63X/1.4 Plan-Apochromat Oil Immersion objective, with Diode 405 nm (DAPI), Argon ion 488 nm (Alexa-488) and DPSS 561 nm (Alexa-555 phalloidin) laser excitation sources, emitted light was filtered using a combination of emission filters and imaged onto Airy detector array producing an effective lateral resolution of ~100 nm. All the images were Airyscan processed with Zen Black Edition (Zeiss Software).

### Assessment of vaccine side effects

To standardize the evaluation of vaccine-associated side effects, we developed a composite scoring framework termed the Side Effect Severity Score (SESS) adapted from the vaccine side effect guideline described elsewhere^59^. Each reported symptom was first categorized by type and severity (local, systemic, or severe) according to previously established criteria for vaccine reactogenicity. For each donor and vaccine dose, symptoms were graded as: 0 = none; 1 = mild (e.g., injection site pain, mild fatigue, or a single local symptom); 2 = moderate (systemic but not severe, e.g., fever, chills, headache, myalgia, or multiple mild symptoms); or 3 = severe (multiple systemic symptoms, prolonged recovery >2 days, swelling requiring medical review, dose-limiting reaction, or hospitalization). To account for compounded burden, additional multipliers were applied: +1 if multiple symptoms occurred at the same dose, +1 if symptoms recurred across multiple doses, and +1 if symptom duration exceeded 2 days (if reported). Scores were then summed to generate an overall SESS per participant, which was categorized as: 0 (no side effects), 1 – 2 (mild), 3 – 4 (moderate), and ≥ 5 (severe). The scores are summarized in **Supplementary Table 21**.

### RNA Sequencing

We performed bulk RNAseq in a total of 21 samples of PBMCs (n = 10 HLA-A*03:01^+^, n = 11 HLA-A*03:01^−^) and compared the two groups based on *HLA* genotype. Total RNA libraries were prepared and sequenced by Novogene Corporation using Illumina NovaSeq platforms following standard protocols^60^. RNA quantity and integrity were assessed to ensure a minimum RNA Integrity Number (RIN) of > 3.0. The raw sequence data generated by Novogene met strict quality criteria as described by the provider^60,61^.

### RNAseq data processing and analysis

We analyzed all 21 donors from an independent cohort which included individuals of European and non-European ancestries. Samples were sequenced in two batches, with raw RNA-seq data merged and then normalized for batch effects (**Supplementary Figure 16**) using Combat-seq^62^ These sequences were then analyzed using the nf-core/rnaseq pipeline (version 3.19.0), executed through Nextflow^63^. Initial quality control was performed with FastQC, followed by adapter and low-quality base trimming using Trim Galore. Reads were then aligned to the reference genome [Human Genome Assembly GRCh38.p14] using STAR. Transcript quantification was accomplished using Salmon, as defined by the pipeline configuration. The resulting gene-level count matrices were imported into R for normalization and differential expression analysis using the DESeq2 package^64^. Additional downstream analyses, including gene set enrichment analysis, were conducted using standard R workflows.

### Statistical analysis

*HLA* associations: In our discovery cohort, we examined the association of five *HLA* loci (*HLA-A, -B, -C, -DRB1, -DQB1*). Data analysis included the first two fields of the allele name as described in the HLA nomenclature, representing the complete molecule at polypeptide sequence resolution. We calculated allele frequencies for all the *HLA* loci, haplotype frequencies using Haplostats R package^65^ and R2 for Linkage Disequilibrium (from our in-house script) between all the pair of loci (**Supplementary Table 14**). We employed a generalized logistic regression model using ‘glm’ in the R (V 4.3) base package to consider relevant covariates, including sex and age. For the replication cohort, we tested only the allele of interest, using the generalized logistic regression model framework as described. We utilized our in-house Python script to construct forest plots. We conducted an analysis of variance (ANOVA) to assess the statistical significance of differences in antibody positivity rates across groups with different counts of HLA-A*03:01 allele(0,1 or 2).

All additional analyses were performed in GraphPad Prism v10.1. Data are shown as mean ± SD, with symbols representing individual donors. Paired comparisons across time points were assessed using two-tailed Wilcoxon matched-pairs tests, and unpaired comparisons between HLA-A*03:01^+^ and HLA-A*03:01^−^ groups by two-tailed Mann–Whitney U tests. For the CBA, cytokine values were background-subtracted from unstimulated or controls; analytes below detection limits were excluded. Correlations between cytokine levels and side-effect severity were evaluated using Spearman’s r. Frequencies of cytokine-producing or tetramer-positive T cells and phagocytic scores were compared using nonparametric tests. All tests were two-tailed; p < 0.05 was considered significant.

## Supplementary Material

Supplementary Files

This is a list of supplementary files associated with this preprint. Click to download.

- Supp2FiguresA3FIN.pdf
- SupplementarytablesA3FIN.xlsx
- Supp1FiguresA3FIN.pdf
